# Inappropriate Implantable Cardioverter-Defibrillator Therapy During Total Hip Arthroplasty

**DOI:** 10.7759/cureus.38973

**Published:** 2023-05-13

**Authors:** Alexander M DeLeon, Rachael Lu, Liting Chen, Yogen Asher

**Affiliations:** 1 Anesthesiology, Northwestern University Feinberg School of Medicine, Chicago, USA

**Keywords:** pacemaker, implantable cardioverter-defibrillator, emi, electromagnetic interference, electrocautery

## Abstract

Electromagnetic interference (EMI) is a known risk factor for triggering inappropriate therapy from implantable cardioverter-defibrillators (ICDs). Recommendations from the American Society of Anesthesiologists focus on EMI when using monopolar electrocautery for supraumbilical surgeries. Infraumbilical surgeries are not considered high risk for EMI; thus, no magnet must be applied routinely to prevent inappropriate ICD therapy intraoperatively. We describe a case of a 71-year-old woman who presented for left total hip arthroplasty with a history of an ICD. The patient's history was significant for non-ischemic cardiomyopathy. Monopolar electrocautery was used, and the level of the surgery was below the umbilicus. She experienced nine inappropriate ICD therapies intraoperatively with no long-term sequelae. The location of the electrocautery dispersion pad may have contributed to inappropriate therapies. Thus, the dispersion pad location should be considered when deciding whether to suspend anti-tachycardia functions intraoperatively. We present a case of inappropriate therapy from an ICD and make a recommendation for preventing such events.

## Introduction

In 2020 the American Society of Anesthesiologists (ASA) published a practice advisory for the perioperative management of patients with cardiac implantable electronic devices [1]. The advisory aimed to facilitate the effective management of electronic devices during surgery and reduce adverse outcomes. The practice advisory cited observational data indicating that implantable cardioverter-defibrillators (ICDs) in the pectoral region were more likely to experience electromagnetic interference (EMI) when surgery was performed above the umbilicus [2]. The consultants agreed, and ASA members were equivocal on whether to suspend anti-tachycardia functions for surgery inferior to the umbilicus. The final recommendation is to suspend anti-tachycardia functions for supraumbilical surgery. The advisory recommendations include "avoiding the indiscriminate use of a magnet over an implantable cardioverter-defibrillator," which could theoretically result in an "R on T" phenomenon leading to a malignant arrhythmia [1].

We present a case of a patient with an ICD with a generator located in the left pectoral region who underwent left total hip arthroplasty and experienced inappropriate ICD therapy. This report highlights the risk of EMI despite following recommended guidelines for the intraoperative management of ICDs.

## Case presentation

**Figure 1:**
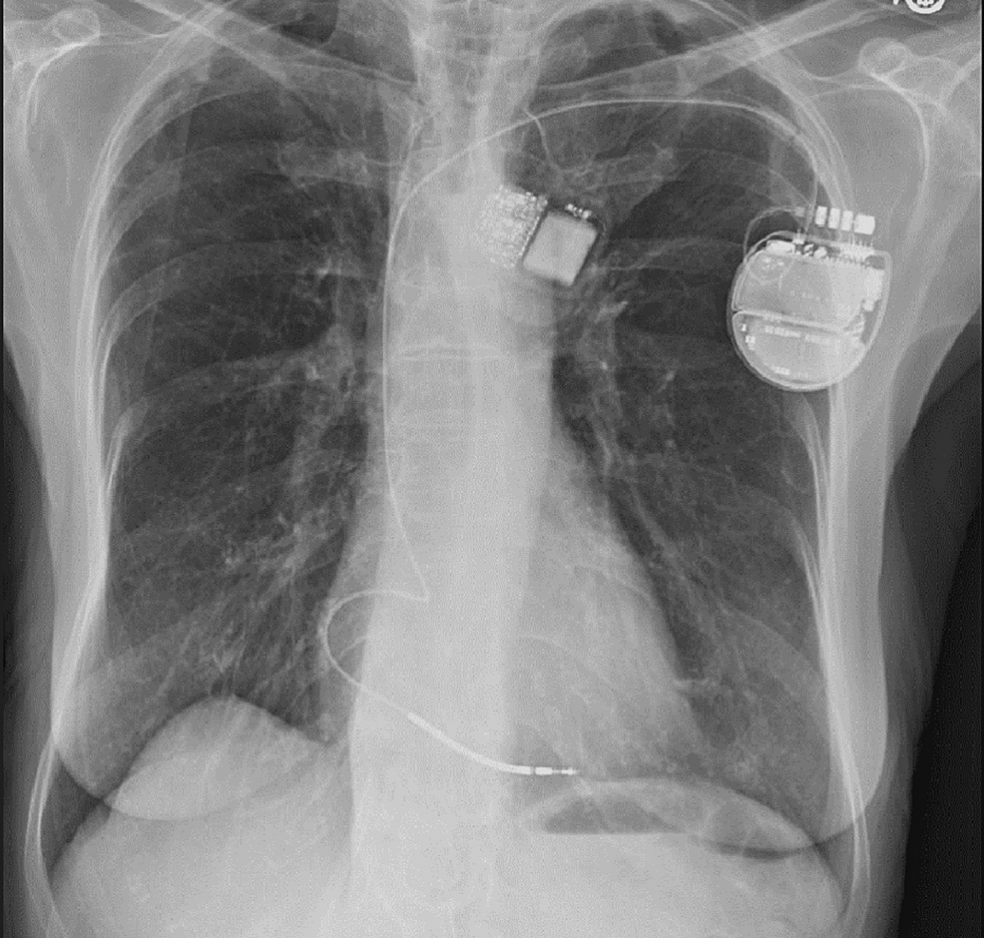
Chest radiograph The radiograph indicates the location of the ICD generator

A 71-year-old woman presented for left total hip arthroplasty. The patient's medical history was significant for a ventricular tachycardia arrest leading to her diagnosis of non-ischemic cardiomyopathy (NICM) two years before surgery. The patient had a single lead right ventricular St. Jude Medical Inc. (Little Canada, Minnesota) implant placed during her NICM diagnosis (Figure 1). Shortly after discharge, she experienced an episode of syncope, and her ICD was noted to have been discharged 28 times. Subsequently, she was found to be in atrial fibrillation with a rapid ventricular rate and, thus, underwent an atrial fibrillation ablation. Since that event, the patient had not received any additional shocks before presenting for total hip arthroplasty surgery with possible hardware removal.

One month before surgery, the patient's ICD was remotely interrogated. The interrogation indicated three runs of non-sustained ventricular tachycardia (NSVT) during the prior year with no evidence of atrial fibrillation or additional shocks. The patient's pacemaker function indicated less than 1% pacing.

The patient was physically active before surgery, tolerating greater than four METS, yet was limited by hip pain. Her laboratory studies were significant only for a Hb of 11.8 g/dL, which was stable. Her most recent ejection fraction was 47% less than two months before surgery. Her medications included amiodarone, buspirone, apixaban, and metoprolol. The patient's vital signs were within normal limits; her height was 5'9", her weight was 64.4 kg, and her BMI was 20.96 kg/m2.

The anesthetic team planned general anesthesia with endotracheal intubation and placing a post-induction arterial pressure catheter. Monopolar electrocautery was used throughout the surgery. The polyhesive adult-size patient return electrode (Valleylab, Covidien, Minneapolis, MN) was placed on the patient's back at the level of the umbilicus. Both coagulation and cutting settings were set to 45.

No magnet was placed due to the infra-umbilical location of the surgery. No episodes of ventricular fibrillation nor ventricular tachycardia were noted on the ECG monitor at any time during the surgery. The patient's heart rate was between 70 and 80 bpm intraoperatively, with her mean arterial blood pressure maintained at greater than 65 mmHg with a phenylephrine infusion no higher than 50 mcg/min. Her rhythm was sinus throughout the procedure.

**Figure 2:**
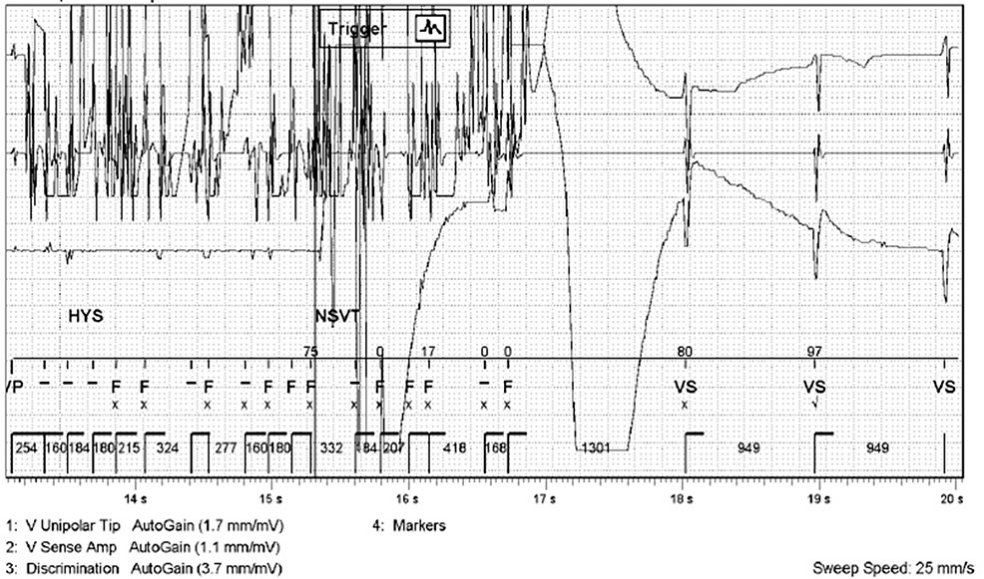
Pacemaker interrogation report The figure illustrates one NSVT event that triggered an inappropriate implantable ICD therapy

The patient's intracardiac device was remotely interrogated six days after surgery. The remaining battery life was 4.1 years compared to 5.6 one month before surgery. The patient was noted to have had nine shocks and four anti-tachycardia pacing events, all of which occurred during the surgery. Her event monitor indicated 30 ventricular fibrillation zone episodes and 28 NSVT episodes. The cardiologist noted that based on the information recorded by the ICD, the event was "suggestive of oversensing of EMI noise, all therapies inappropriate, and no true ventricular arrhythmias detected" (Figure 2). Each therapeutic shock delivered 30 J, and the ventricular fibrillation rates at the time of each shock were noted to be 240 to 480 bpm. The patient's subsequent hospital and postoperative course proceeded uneventfully.

## Discussion

Based on the ASA Practice Advisory [1], no magnet was needed to suspend anti-tachycardia therapy during this surgery due to the infra-umbilical location of the surgery. This case represents a rare occurrence of documented inappropriate discharge of an ICD despite adherence to a national practice advisory for surgeries located below the umbilicus.

Singleton et al. [3] published two cases of inappropriate ICD therapy despite below the umbilicus surgical sites. Those cases both involved underbody electrosurgery dispersive electrodes. The dispersive electrodes described (Ethicon Megadyne, Johnson & Johnson, New Brunswick, NJ) measured 36 by 20 inches. The authors describe the position of the dispersive pads, which extended above the umbilicus. Singleton et al. [3] recommended that providers consider factors other than surgical location when making magnet-use decisions. In addition, they recommended noting the distance to the sensing lead rather than the generator and considering short stature a risk for inappropriate ICD therapy [3]. Finally, they mentioned that the type of dispersion pad (i.e., underbody) should be considered when deciding if a magnet is necessary to suspend anti-tachycardia functions.

Other cases of underbody dispersion pads associated with inappropriate ICD therapy have been reported [4,5]. As proposed by Singleton et al. [3], the current may have traveled close enough to the sensing electrode despite a far distance to the pacemaker generator. Thus, the EMI may have traveled to the sensing electrode of the patient's ICD.

Our case and the other cited cases indicate that the ASA Practice Advisory should consider additional factors other than the location of the surgery when monopolar electrocautery is utilized [3-5]. Our case is unique in that it illustrates that the recommendations of Singleton et al. [3], including distance to the sensing electrode, patient height, and underbody dispersion electrode, may not be enough. Our patient's height was 5'9" (i.e., not short stature), her sensing electrode was distant from the surgical site, and the dispersion electrode was not an underbody electrode.

## Conclusions

When a patient has an ICD and is undergoing surgery below the umbilicus, we recommend placing the dispersion pad below the umbilicus (e.g., contralateral leg for a total hip arthroplasty). If such placement is impractical, a magnet may be used to suspend the anti-tachycardia functions. If the patient is also pacemaker dependent, and the dispersion pad is placed at or above the umbilicus, reprogramming to an asynchronous mode should be considered with anti-tachycardia functions suspended.
